# Effective, Broad Spectrum Control of Virulent Bacterial Infections Using Cationic DNA Liposome Complexes Combined with Bacterial Antigens

**DOI:** 10.1371/journal.ppat.1000921

**Published:** 2010-05-27

**Authors:** Robin Ireland, Norma Olivares-Zavaleta, Jonathan M. Warawa, Frank C. Gherardini, Clayton Jarrett, B. Joseph Hinnebusch, John T. Belisle, Jeffery Fairman, Catharine M. Bosio

**Affiliations:** 1 Laboratory of Intracellular Parasites, Rocky Mountain Laboratories, NIAID, NIH, Hamilton, Montana, United States of America; 2 Laboratory of Zoonotic Pathogens, Rocky Mountain Laboratories, NIAID, NIH, Hamilton, Montana, United States of America; 3 Department of Microbiology, Immunology and Pathology, Colorado State University, Fort Collins, Colorado, United States of America; 4 Juvaris Biotherapeutics, Burlingame, California, United States of America; Midwest Research Institute, United States of America

## Abstract

Protection against virulent pathogens that cause acute, fatal disease is often hampered by development of microbial resistance to traditional chemotherapeutics. Further, most successful pathogens possess an array of immune evasion strategies to avoid detection and elimination by the host. Development of novel, immunomodulatory prophylaxes that target the host immune system, rather than the invading microbe, could serve as effective alternatives to traditional chemotherapies. Here we describe the development and mechanism of a novel pan-anti-bacterial prophylaxis. Using cationic liposome non-coding DNA complexes (CLDC) mixed with crude *F. tularensis* membrane protein fractions (MPF), we demonstrate control of virulent *F. tularensis* infection *in vitro* and *in vivo*. CLDC+MPF inhibited bacterial replication in primary human and murine macrophages *in vitro*. Control of infection in macrophages was mediated by both reactive nitrogen species (RNS) and reactive oxygen species (ROS) in mouse cells, and ROS in human cells. Importantly, mice treated with CLDC+MPF 3 days prior to challenge survived lethal intranasal infection with virulent *F. tularensis*. Similarly to *in vitro* observations, *in vivo* protection was dependent on the presence of RNS and ROS. Lastly, CLDC+MPF was also effective at controlling infections with *Yersinia pestis*, *Burkholderia pseudomallei* and *Brucella abortus*. Thus, CLDC+MPF represents a novel prophylaxis to protect against multiple, highly virulent pathogens.

## Author Summary

Conventional treatment of bacterial infections typically includes administration of antibiotics. However, many pathogens have developed spontaneous resistance to commonly used antibiotics. Development of new compounds that stimulate the host immune system to directly kill bacteria by mechanisms different from those utilized by antibiotics may serve as effective alternatives to antibiotic therapy. In this report, we describe a novel compound capable of controlling infections mediated by different, unrelated bacteria via the induction of host derived reactive oxygen and reactive nitrogen species. This compound is comprised of cationic liposome DNA complexes (CLDC) and crude membrane preparations (MPF) obtained from attenuated *Francisella tularensis* Live Vaccine Strain (LVS). Pretreatment of primary mouse or human cells limited replication of virulent *F. tularensis, Burkholderia pseudomallei, Yersinia pestis* and *Brucella abortus in vitro*. CLDC+MPF was also effective for controlling lethal pulmonary infections with virulent *F. tularensis*. Thus, CLDC+MPF represents a novel antimicrobial for treatment of lethal, acute, bacterial infections.

## Introduction

Historically, control of bacterial infections has been dependent on administration of antibiotics. However, with many acute, lethal, bacterial infections, e.g. those mediated by *Francisella tularensis, Yersinia pestis* and *Staphylococcus aureus*, diagnosis and timely administration of appropriate antibiotics represents a significant hurdle in successful treatment of disease mediated by these pathogens. Further, mass administration of prophylactic antibiotics in an outbreak situation may result in the generation of antibiotic resistant strains, rendering this treatment ineffective for both ongoing infections and future outbreaks. Thus, there is a need for novel, broad spectrum, prophylaxis against highly pathogenic bacterial infections.

*F. tularensis* is a Gram negative, facultative intracellular bacterium and the causative agent of Tularemia. *F. tularensis* is extremely infectious, capable of causing acute, lethal, disease following inhalation of as few as 10–15 bacteria [1], [2]. Currently, there is no vaccine approved for use against *F. tularensis*. Although antibiotic therapy can successfully treat pneumonic Tularemia, therapy must be initiated within the first few days following the onset of symptoms when individuals are often unaware of the severity of their infection [3]. Furthermore, treatment with antibiotics can fail to adequately clear *F. tularensis*, resulting in recrudescence of infection once antibiotic therapy ends [4], [5], [6].

A number of studies have described development of novel anti-microbials that target the host immune response rather than the invading pathogen [7], [8], [9]. These immunotherapeutics target host pathways which either directly activate effector cells or relieve pathogen induced suppression of host killing mechanisms, resulting in control and elimination of a wide variety of microorganisms. In the case of intracellular pathogens such as *F. tularensis*, targeting host effector mechanisms is appealing since some antibiotics preferred for treatment of Tularemia, e.g. gentamicin, poorly permeate the host cell and therefore fail to reach the targeted organism. Activation of host effector cells capable of killing intracellular pathogens with novel immunotherapeutics or prophylaxes represents a viable alternative, or supplement, to exiting chemotherapy.

In this report we describe a novel anti-microbial comprised of cationic liposome DNA complexes (CLDC) and crude membrane protein fraction (MPF) derived from attenuated *F. tularensis* strain LVS. CLDC+MPF effectively controlled *in vivo* and *in vitro* infections of virulent *F. tularensis* strain SchuS4 in mouse and human cells, respectively. The combined delivery of CLDC and MPF was critical for mediating this protection, since treatment with CLDC or MPF alone failed to attenuate *F. tularensis* replication and pathogenicity. The dramatic control of *F. tularensis* infection mediated by CLDC+MPF was dependent on stimulation of both reactive oxygen and nitrogen species (ROS and RNS, respectively) *in vivo* and *in vitro*. Finally, we demonstrate that CLDC+MPF was also an effective antimicrobial against three other important bacteria, *Burkholderia pseudomallei, Yersinia pestis* and *Brucella abortus*. Thus, data presented herein represents an important step toward development of novel, efficacious, broad spectrum, antimicrobial therapy directed against highly pathogenic microbes.

## Results

### CLDC+MPF controlled replication of SchuS4 in mouse and human cells

To establish the anti-microbial potential of CLDC+MPF we first examined the effect of this compound on the infection and replication of SchuS4 in mouse and human macrophages. Cells were treated with either 5% dextrose water (D5W;untreated), CLDC, MPF or CLDC+MPF approximately 18 h prior to SchuS4 infection. At the indicated time points, viable intracellular bacteria were enumerated. Macrophages treated with MPF or CLDC alone failed to control SchuS4 replication (Table 1 and 2). Additionally, treatment of mouse macrophages with MPF or CLDC alone exacerbated SchuS4 replication in these cells (Table 1). In contrast to cells treated with the individual components or untreated controls, cells pre-treated with CLDC+MPF had significantly fewer intracellular bacteria (Table 1 and 2). It was possible that treatment with CLDC+MPF induced cell death which in turn resulted in smaller numbers of intracellular SchuS4. However, staining by trypan blue revealed that, similar to untreated controls, greater than 90% of CLDC+MPF treated macrophages were viable at the time of infection (Figure S1). Thus, generalized cell death could not account for the reduction in bacterial loads following CLDC+MPF treatment. Together this data shows that while CLDC+MPF did not limit uptake of SchuS4, it did control replication of the intracellular bacterium.

**Table 1:** CLDC+MPF controls replication of *F. tularensis* SchuS4 in mouse macrophages.

Treatment	Time after Infection (hours)	CFU/ml (log10)±SEM
untreated	3	2.13±0.09
CLDC	3	3.16±0.13
MPF	3	3.34±0.13
CLDC+MPF	3	3.10±0.09
untreated	24	3.30±0.23
CLDC	24	5.30±0.01
MPF	24	4.72±0.19
CLDC+MPF	24	1.60±0.35

**Table 2:** CLDC+MPF controls replication in *F. tularensis* SchuS4 in human macrophages.

Treatment	Time after Infection (hours)	CFU/ml (log10) ±SEM
untreated	3	2.84±0.09
CLDC	3	2.76±0.03
MPF	3	3.01±0.02
CLDC+MPF	3	2.49±0.01
untreated	24	4.88±0.05
CLDC	24	4.56±0.09
MPF	24	4.61±0.02
CLDC+MPF	24	3.42±0.30

**Figure 1:**
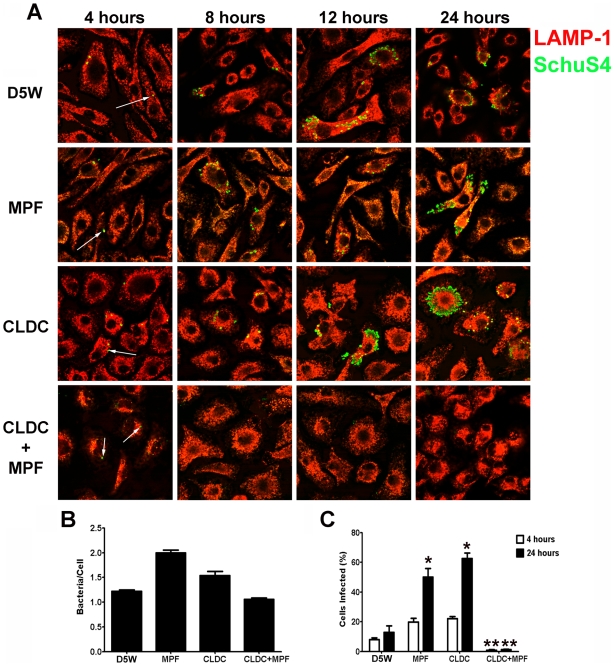
CLDC+MPF mediated control of *F. tularensis* replication in mouse macrophages. Mouse macrophages were treated with D5W (untreated), MPF, CLDC, or CLDC+MPF 18 h prior to infection. Four, 8, 12 and 24 h after infection phagocytosis and intracellular replication of *F. tularensis* was monitored by microscopy. Exposure to CLDC, MPF or CLDC+MPF did not impact the number of cells infected (A and C) nor the number of bacteria entering each cell (B). MPF and CLDC treatment alone significantly increased the number SchuS4 infected cells 24 h after infection compared to untreated controls (* = p<0.01) (A and C). In contrast, CLDC+MPF treated cultures had fewer infected cells within 8 h of infection through 24 h after infection (** = p<0.01) (A and C). White arrows indicate intracellular SchuS4. Data is representative of four experiments. Error bars represent SEM.

**Figure 2 ppat-1000921-g002:**
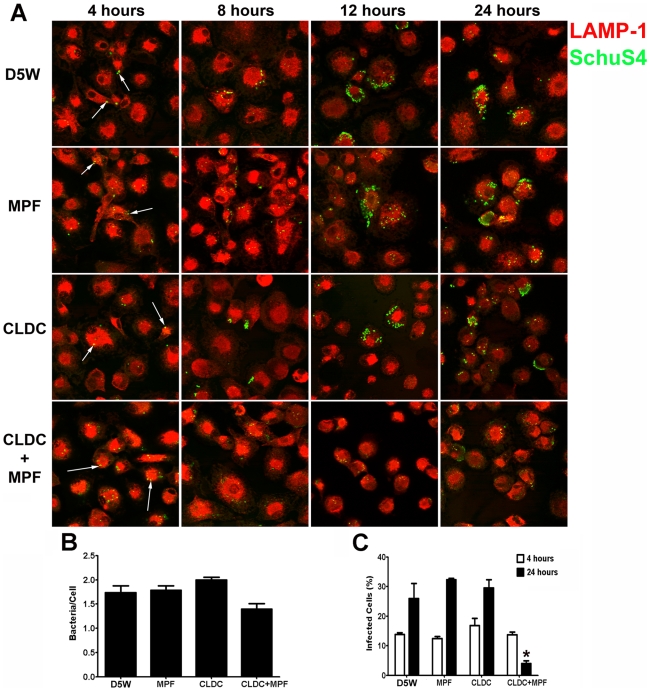
CLDC+MPF mediated control of *F. tularensis* replication in human macrophages. Human macrophages were treated with D5W (untreated), MPF, CLDC, or CLDC+MPF 18 h prior to infection. Four, 8, 12 and 24 h after infection phagocytosis and intracellular replication of *F. tularensis* was monitored by microscopy. Exposure to CLDC, MPF or CLDC+MPF did not impact the number of cells infected (A and C) nor the number of bacteria entering each cell (B). CLDC+MPF treated cultures had fewer infected cells within 12 h of infection and through 24 h after infection (* = p<0.01) (A and C). White arrows indicate intracellular SchuS4. Data is representative of four experiments. Error bars represent SEM.

Intracellular replication of SchuS4 in macrophages is dependent on their ability to escape the phagolysosome within the first hour of infection [10]. Thus, although CLDC+MPF did not appear to alter the initial uptake of SchuS4 by macrophages, it was possible that this mixture inhibited the ability of SchuS4 to escape the phagolysosome subsequently resulting in killing of the bacterium. Phagosomal escape by SchuS4 can be measured by microscopy by the loss of LAMP-1 colocalization with the bacterium following macrophage phagocytosis [10]. To assess the effect CLDC, MPF and CLDC+MPF had on SchuS4 phagosomal escape we next examined co-localization of SchuS4 with LAMP-1 in treated mouse and human macrophages 24 h after infection by microscopy. As described above, CLDC+MPF did not significantly alter the number of bacteria phagocytosed by either mouse or human macrophages compared to untreated controls (Figure 1B and 2B, respectively). Further, CLDC+MPF treatment did not inhibit the ability of SchuS4 to escape into the cytosol of infected cells as evident by the absence of SchuS4 co-localization with LAMP-1 4 hours after infection in all treatment groups (Figure 1A and 2A). In contrast, CLDC+MPF significantly reduced the number of infected human and mouse macrophages 24 h after infection (Figures 1A, C and 2A, C, respectively). We also examined if CLDC+MPF mediated killing of SchuS4 by electron microscopy. As expected, in untreated cells SchuS4 was present as intact bacteria in the cytosol (Figure S2). In contrast, bacteria present in CLDC+MPF treated macrophages were largely degraded with few to no intact bacteria present in the cytosol (Figure S2).

Following entry and escape into the host cytosol, SchuS4 undergoes a lag period of approximately 4 hours prior to initiation of replication. Replication in the primary infected cell then commences and continues over a 12–18 hour time period [11]. Thus, we next determined at what point after infection CLDC+MPF could restrict intracellular SchuS4 growth in macrophages, Cultures treated with CLDC+MPF 4 hours after infection had significantly fewer infected cells compared to untreated controls (p<0.05) (Figure S3). In contrast, macrophages treated with CLDC+MPF 12 hours after SchuS4 infection failed to control bacterial replication compared to untreated controls (Figure S3). Together, these data suggested that in order for CLDC+MPF to exert its protective effects macrophages must be stimulated at time points prior to intracellular replication of SchuS4.

### CLDC+MPF mediated induction of ROS and RNS

**Figure 3 ppat-1000921-g003:**
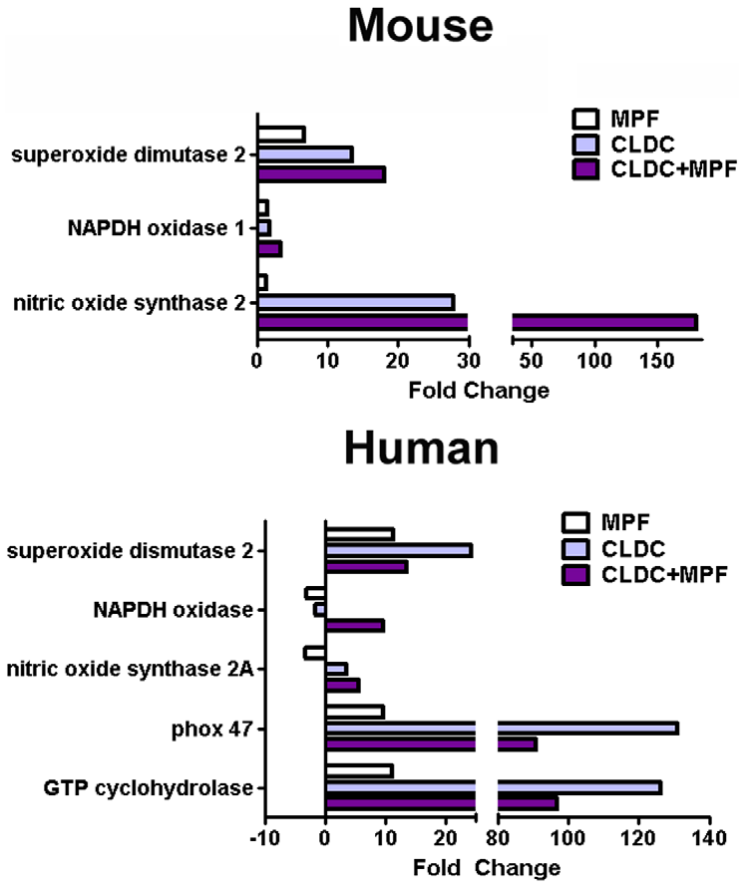
Induction of RNS and ROS genes by CLDC+MPF. Mouse and human macrophages were treated with D5W, CLDC, MPF or CLDC+MPF. After 16 and 12 h, respectively, gene expression was monitored by quantitative RT-PCR. Change in the expression of the indicated genes is represented as fold change over D5W treated controls. Data is representative of three experiments.

Stimulation of ROS and RNS in mammalian cells represents an important mechanism by which the host controls and eliminates bacterial growth, especially in the intracellular environment (as reviewed, [12]). Furthermore, ROS and RNS have been implicated as important mediators for control of attenuated strains of *F. tularensis* [13], [14], [15], [16]. Thus, we hypothesized that CLDC+MPF may be mediating control of SchuS4 infection in macrophages via induction of ROS and/or RNS. We first determined if MPF, CLDC, or CLDC+MPF induced expression of genes associated with oxidative stress. Mouse macrophages treated with CLDC+MPF had higher expression levels of genes associated with RNS (nitric oxide synthetase 2) and ROS (superoxide dismutase 2, NADPH oxidase 1) compared to untreated controls (Figure 3). Induction of RNS and ROS related genes was highest when cells were treated with CLDC+MPF rather than CLDC or MPF alone (Figure 3). Similarly, treatment of human cells with CLDC+MPF resulted in increased expression of genes associated with generation of ROS, e.g. phox p47, superoxide dismutase 2, NADPH oxidase, and GTP cyclohydrolase, as well as RNS, e.g. and nitric oxide synthase 2A compared to untreated cells and cells treated with MPF alone (Figure 3). Interestingly, although CLDC alone failed to control SchuS4 infection in human macrophages, cells treated with this compound in the absence of MPF had greater gene transcription for three genes involved in generation of ROS, i.e. superoxide dismutase 2, phox47 and GTP cyclohydrolase compared to cells treated with CLDC+MPF (Figure 3). We also compared induction of ROS and RNS related genes in cells treated with a known inducer of RNS, IFN-γ to CLDC+MPF. CLDC+MPF elicited higher gene transcription of nitric oxide synthetase (nos2), NADPH oxidase and superoxide dismutase (sod2) in mouse cells and GTP cyclohydrolase, nitric oxide synthetase 3 (nos3), NADPH oxidase, phox 47 and sod2 in human cells than IFN-γ (Figure S4).

**Figure 4 ppat-1000921-g004:**
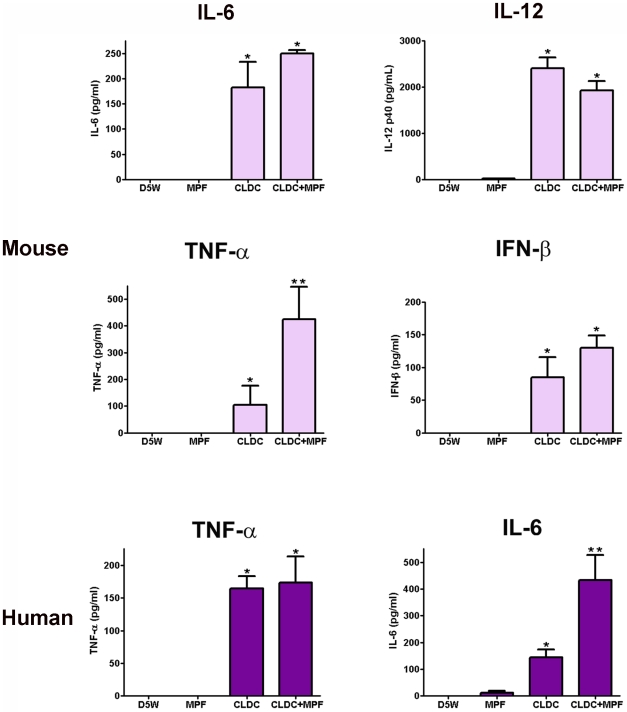
CLDC+MPF induced production of pro-inflammatory cytokines in macrophages. Mouse and human macrophages were treated with D5W (untreated), MPF, CLDC, or CLDC+MPF and 24 h later supernatants were assessed for cytokines by ELISA. In mouse cells, CLDC and CLDC+MPF induced significantly more IL-6, IL-12 and IFN-β compared to D5W or MPF treated cells (* = p<0.01). Whereas, CLDC+MPF induced significantly more TNF-α compared to all other groups (** = p<0.01). In human cells CLDC and CLDC+MPF induced significantly more TNF-α compared to D5W or MPF treated cells (* = p<0.01). Whereas CLDC+MPF induced significantly more IL-6 compared to all other groups (** = p<0.01). Data is representative of four experiments. Error bars represent SEM.

Induction of both RNS and ROS is often dependent on the presence of IFN-γ, TNF-α, IFN-β and other pro-inflammatory cytokines. In correlation with the expression levels of RNS and ROS associated genes, mouse macrophages treated with CLDC+MPF secreted significantly higher concentrations of TNF-α compared to cells treated with CLDC or MPF alone (Figure 4). Addition of MPF to CLDC also increased secretion of IL-6 and IFN-β from mouse cells compared to cells treated with CLDC alone, however these differences were not significant (Figure 4).

We also monitored production of cytokines from human cells treated with MPF, CLDC or CLDC+MPF. Although no IL-12p40 or IFN-β was detected in any cell culture supernatant, CLDC+MPF induced secretion of significantly higher concentrations of IL-6 from human macrophages compared to cells treated with CLDC alone (Figure 4). Human macrophages also produced significantly higher concentrations of TNF-α in response to CLDC and CLDC+MPF compared to cells treated with D5W or MPF (Figure 4). Thus, both CLDC and CLDC+MPF elicited production of cytokines associated with induction of RNS and ROS, and in some cases, the combination of CLDC+MPF resulted in higher concentrations of these cytokines.

### CLDC+MPF mediated control of SchuS4 was dependent on ROS and RNS *in vitro*

**Figure 5:**
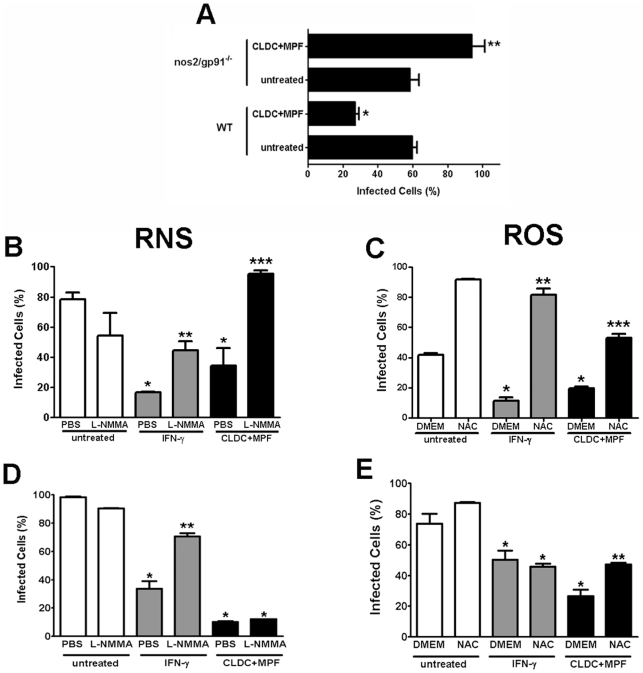
CLDC+MPF control of *F. tularensis* is dependent on RNS and ROS. (A) Macrophages from wild type or nos2/gp91^−/−^ mice were treated with D5W (untreated) or CLDC+MPF for 18 h followed by infection with SchuS4. Intracellular replication was monitored by microscopy and the percent infected cells is depicted for each group. CLDC+MPF significantly reduced the number of SchuS4 infected wild type cells (* = p<0.01), whereas CLDC+MPF treatment of nos2/gp91^−/−^ significantly increased the number of infected macrophages (** = p<0.01). (B and C) Wild type mouse macrophages were treated with D5W (untreated), IFN-γ or CLDC+MPF in the presence or absence of L-NMMA (B) or NAC (C) and then infected with SchuS4. IFN-γ and CLDC+MPF significantly lowered SchuS4 infected cells (* = p<0.05) compared to untreated controls. Addition of L-NMMA and NAC significantly increased the number of infected macrophages compared to IFN-γ (** = p<0.05) or CLDC+MPF (*** = p<0.01) treated controls. (D and E) Human macrophages were treated with D5W (untreated), IFN-γ or CLDC+MPF in the presence or absence of L-NMMA (D) or NAC (E) and then infected with SchuS4. IFN-γ and CLDC+MPF significantly lowered SchuS4 infected cells (* = p<0.05) compared to untreated controls. Addition of L-NMMA significantly increased the number of infected macrophages compared to IFN-γ (** = p<0.05), but not CLDC+MPF treated controls. Addition of NAC significantly increased the number of infected macrophages compared to CLDC+MPF (** = p<0.01), but not IFN-γ, treated controls.

To determine if the induction ROS, RNS or both ROS and RNS by treatment of cells with CLDC+MPF contributed to control of SchuS4 infection, we first examined the ability of CLDC+MPF to limit SchuS4 replication 24 h after infection in macrophages obtained from mice deficient for both RNS and ROS. There was not a difference in the percentage of infected cells among untreated wild type and nos2/gp91^−/−^ macrophages, suggesting that nos2/gp91^−/−^ cells were not more susceptible to SchuS4 infection compared to wild type cells (Figure 5A). As previously observed, wild type macrophages treated with CLDC+MPF had significantly fewer infected macrophages compared to untreated cells (p<0.01) (Figure 5A). In contrast, CLDC+MPF failed to control SchuS4 infection in macrophages from nos2/gp91^−/−^ mice (Figure 5A). Furthermore, pre-treatment of nos2/gp91^−/−^ macrophages with CLDC+MPF resulted in significantly more infected macrophages 24 h after the onset of the experiment (p<0.01) (Figure 5A). Thus, CLDC+MPF mediated control of SchuS4 infection was at least partially dependent on ROS and RNS in mouse macrophages.

To determine the contribution of RNS and ROS in CLDC+MPF mediated control of SchuS4 infection in wild type mouse macrophages, we conducted additional experiments using compounds that specifically interfere with either the generation of RNS (L-NMMA) or ROS (NAC). IFN-γ has been shown to mediate killing of intracellular bacteria, including Francisella, following stimulation of both ROS and RNS (23). Thus, macrophages pretreated with IFN-γ followed by exposure to L-NMMA or NAC served as positive controls for inhibition of ROS and RNS species. As expected, pretreatment of mouse and human macrophages with IFN-γ significantly reduced the number of cells infected with SchuS4 compared to untreated controls (p<0.01) (Figure 5B–E). The role of both RNS and ROS in IFN-γ mediated control of SchuS4 in mouse macrophages was confirmed by significant increases in SchuS4 infected cells following addition of either L-NMMA or NAC to IFN-γ treated cells (p<0.05) (Figure 5B–C). Similarly, addition of L-NMMA or NAC to CLDC+MPF pretreated mouse macrophages reversed the protective effect observed with this prophylaxis (Figure 5B–C). Furthermore, addition of either L-NMMA or NAC to CLDC+MPF treated cells also appeared to increase the number of infected cells over untreated controls (Figure 5B–C). Thus, in mouse macrophages CLDC+MPF mediated killing of SchuS4 was dependent on the generation of both RNS and ROS.

In contrast to mouse cells, inhibition of RNS had little effect on the ability of CLDC+MPF to control SchuS4 infection in primary human macrophages (Figure 5D). Rather, inhibition of ROS following addition of NAC significantly increased the number of infected cells among CLDC+MPF treated samples (p<0.05) (Figure 5E). This suggested that in human macrophages generation of ROS, rather than RNS, following treatment with CLDC+MPF was the primary mechanism for control of SchuS4 in human macrophages.

### CLDC+MPF protected against SchuS4 infection *in vivo*

Macrophages are one of the primary cells targeted by SchuS4 for replication *in vivo* [17]. The dramatic effect CLDC+MPF had on control of intracellular growth of SchuS4 in macrophages *in vitro* (Figures 1–5) suggested that this compound may be an effective anti-microbial *in vivo*. Thus, we next assessed the ability of CLDC, MPF and CLDC+MPF to protect mice from pulmonary challenge with SchuS4. Pretreatment of mice with CLDC alone failed to protect animals from SchuS4 related mortality, regardless of the route of time at which the CLDC was administered. In fact, administration of CLDC within 48 hours prior to infection by any route exacerbated disease, as indicated by an increase in the mean time to death of treated animals compared to untreated controls (Table 3). In contrast, administration of CLDC intravenously, intranasally or intraperitoneally 72 hours prior to infection modestly increased the mean time to death (∼0.2 days) (Table 3). Thus, as observed among in vitro stimulated macrophages, CLDC did alone did not protect animals for death following SchuS4 infection.

**Table 3:** CLDC alone does not protect against pulmonary *F. tularensis* SchuS4 infections^a^.

Treatment	Route	Time Before Challenge(hours)	Survivors/Total	Mean Time to Death(MTD) ±SEM
untreated	-	-	0/10	5.8±0.133
CLDC^b^	IV^c^	24	0/10	5.0±0.000
		48	0/10	5.0±0.000
		72	0/10	6.0±0.000
CLDC	IP^d^	24	0/10	5.4±0.163
		48	0/10	4.0±0.000
		72	0/10	6.0±0.000
CLDC	IN^e^	24	0/10	4.6±0.163
		48	0/10	4.8±0.133
		72	0/10	6.0±0.000
CLDC	SC^f^	24	0/10	5.2±0.133
		48	0/10	5.0±0.000
		72	0/10	4.7±0.213

a Balb/c mice were challenged intranasally with 25 CFU *F. tularensis* SchuS4.

b Cationic DNA Liposome Complexes,

c intravenous,

d intraperitoneal,

e intranasal,

f subcutaneous.

**Figure 6:**
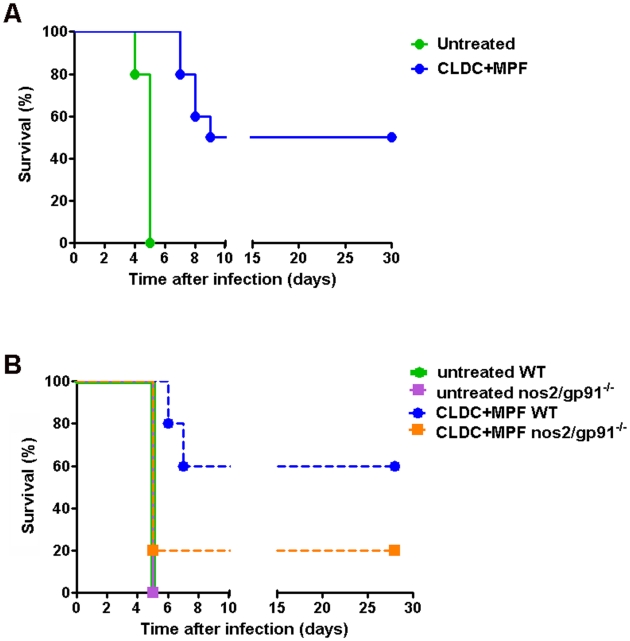
CLDC+MPF enhanced survival of SchuS4 infection *in vivo*. (A) Mice (n = 10 per group) were treated with CLDC+MPF i.v. 3 days prior to intranasal challenge with SchuS4. Significantly more CLDC+MPF treated mice survived infection compared to untreated controls (p = 0.0027). (B) Wild type (WT) or nos2/gp91^−/−^mice (n = 5 per group) were treated with CLDC+MPF 3 days prior to challenge with SchuS4. Sixty percent of CDLC+MPF treated WT mice survived infection, whereas significantly fewer nos2/gp91^−/−^ survived (p = 0.0385). Data are representative of two experiments.

Administration of CLDC three days prior to challenge resulted in a minor increase in mean time to death. Furthermore, treatment of animals prior to that time point resulted in exacerbated disease. Thus, we chose to examine the protective efficacy of CLDC+MPF in animals treated three days prior to infection. The results of these studies are depicted in Table 4. Administration of CLDC+MPF intranasally failed to protect or improve survival of SchuS4 infections. Twenty percent of animals treated with CLDC+MPF subcutaneously or intraperitoneally survived SchuS4 infection. Additionally, animals treated via these routes that succumbed to infection survived longer than untreated controls. Animals treated with CLDC+MPF intravenously had the greatest survival rate, with approximately 50% of these animals surviving SchuS4 infection (Table 4 and Figure 6A). Intravenous injection of either CLDC or MPF failed to protect animals from succumbing to infection (Table 4). Furthermore, intravenous injection of CLDC+MPF 1,2 or 7 days prior to infection failed to protect animals against SchuS4 (data not shown). Thus, in vivo protection against SchuS4 required CLDC and MPF delivered three days prior to infection.

**Table 4:** Effect of delivery route and antigen on CLDC+MPF mediated protection against *F. tularensis* SchuS4^a^.

Treatment^b^	Route	Survivors/Total	Mean Time to Death(MTD) ±SEM
untreated	-	0/5	5.4±0.400
CLDC+MPF^c^	IV^d^	6/10	8.0±0.000
	IP^e^	2/10	6.5±0.267
	IN^f^	0/10	6.0±0.000
	SC^g^	2/10	7.1±0.398
CLDC	IV	0/10	6.2±0.112
MPF	IV	0/10	4.8±0.200
LVS LPS	IV	0/10	5.4±0.245
SchuS4 LPS	IV	0/10	5.8±0.200
CLDC+LVS LPS	IV	0/10	8.0±0.000
CLDC+SchuS4 LPS	IV	0/10	7.8±0.200

a Balb/c mice were challenged intranasally with 25 CFU *F. tularensis* SchuS4.

b Mice were treated 3 days prior to challenge,

c Cationinc DNA Liposome Complexes,

d intravenous,

e intraperitoneal,

f intranasal,

g subcutaneous.

Previous studies have shown that LVS LPS can protect mice from lethal LVS infections [18], [19], [20], [21], [22]. MPF contains LVS LPS. Thus, we postulated that LVS LPS present in MPF may represent an important immunogen for conferring the protection observed in CLDC+MPF treated mice. To test this hypothesis, mice were injected intravenously with LPS purified from LVS alone or in combination with CLDC. Surprisingly, neither LVS LPS alone nor LVS LPS in CLDC protected mice from lethal SchuS4 infection (Table 4). However, LVS LPS in CLDC did increase the mean time to death by approximately 2 days compared to untreated controls (Table 4). This suggested that LVS LPS combined with CLDC was able to stimulate the host immune response for minimal control of SchuS4 infection. Although LPS from LVS and SchuS4 do not elicit strong production of pro-inflammatory cytokines, it was possible that there might be other differences in the immunostimulating potential of these two LPS molecules that would only be revealed in vivo [23], [24], [25]. Thus, we also compared protective efficacy of SchuS4 LPS to protect against SchuS4 infection. Similar to LVS LPS, mice treated with SchuS4 LPS were not protected from death (Table 4). However, inclusion of CLDC did modestly increase the mean time to death in SchuS4+CLDC treated animals (Table 4). Together, these data suggest that Francisella LPS was not the major component of MPF mediating protection against SchuS4 infection in CLDC+MPF treated animals.

Given the importance of both ROS and RNS in CLDC+MPF mediated control of SchuS4 infection in mouse macrophages *in vitro*, we also assessed the role of these antimicrobial host components *in vivo*. Pretreatment of mice with CLDC+MPF protected significantly more wild type animals from SchuS4 compared to untreated controls (p = 0.0027) (Figure 6B). In contrast to the protection observed in wild type animals, CLDC+MPF did not significantly increase the number of surviving nos2/gp91^−/−^ compared to untreated controls (p = 0.3173) (Figure 6B). Furthermore, untreated nos2/gp91^−/−^ mice were not more susceptible to SchuS4 infection compared to untreated wild type mice. This suggested that the lack of protection observed in CLDC+MPF treated nos2/gp91^−/−^ was not due to inherent lack of resistance to *F. tularensis* in these animals. Rather, our data suggests that in the absence of intact pathways for generation of ROS and RNS CLDC+MPF activates cells that render them more susceptible to infection. These results further underscore the importance of induction of ROS and RNS in *F. tularensis* infections. Together this data confirmed that protection mediated by CLDC+MPF was dependent on stimulation of pathways associated with oxidative stress *in vivo*.

### CLDC+MPF protected against multiple bacterial pathogens

**Figure 7:**
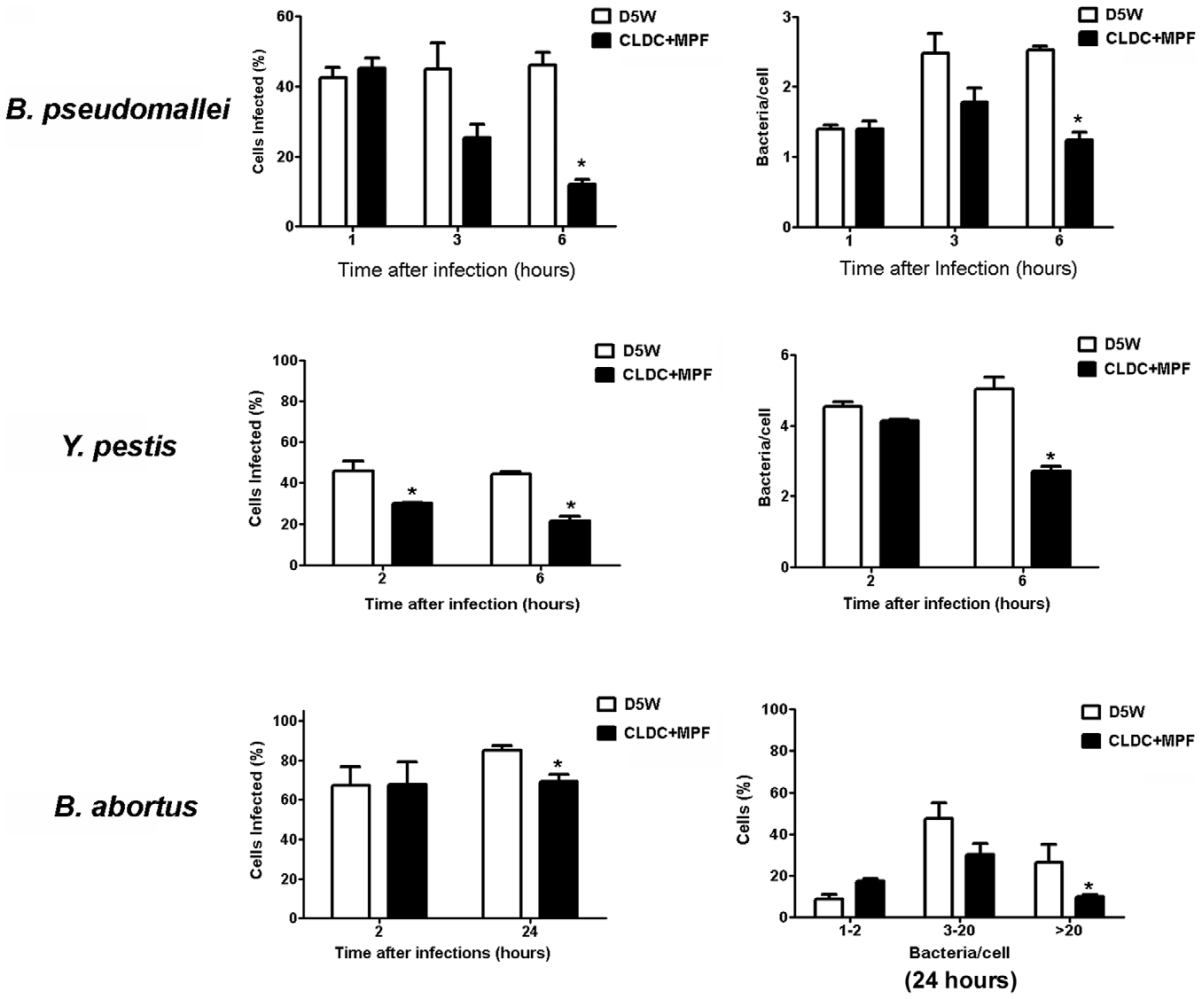
CLDC+MPF mediated protection against unrelated bacterial pathogens. Human macrophages were untreated (D5W) or treated with CLDC+MPF 18 h prior to infection followed by infection with *B. pseudomallei*, *Y. pestis* or *B. abortus*, At the indicated time points cells were analyzed for intracellular bacteria by microscopy. CDLC+MPF significantly reduced the number of infected cells and the number of bacteria per cell regardless of the species of infecting bacteria (* = p<0.05). Data is representative of three experiments. Error bars represent SEM.

Previous experiments have shown that CLDC alone is an effective prophylaxis against *B. pseudomallei* and attenuated strains of Francisella [26], [27]. However, CLDC alone failed to protect against virulent Francisella and *Y. pestis* ([27] and CM Bosio, unpublished data). As shown above, compared to CLDC alone, CLDC+MPF provided superior protection against virulent Francisella (Table 1 and 2; Figures 1 and 2). Thus, it was possible that the combination of CLDC and antigen may also effectively control infections mediated by other, unrelated pathogens. Furthermore, the ability of CLDC or CLDC+MPF to control bacterial infections in human cells has not been examined. To assess the anti-bacterial capabilities of CLDC+MPF, we pretreated human macrophages with CLDC+MPF, infected them with *B. pseudomallei*, *Y. pestis* or *B. abortus*, and evaluated bacterial replication over time. As previously observed with CLDC alone in mouse cells, CLDC+MPF controlled both the number of *B. pseudomallei* infected human macrophages and replication of intracellular bacteria within 6 h of infection (Figure 7). Surprisingly, CLDC+MPF also reduced the number of cells infected with *Y. pestis* at 2 and 6 h after infection and *B. abortus* 24 h after infection (Figure 7). Furthermore, treatment of cells with CLDC+MPF inhibited the intracellular replication of both *Y. pestis and B. abortus* (Figure 7). Together this data suggests that, unlike CLDC alone, stimulation of cells with CLDC+MPF can aid in the control of several different, unrelated, virulent bacteria.

## Discussion

The discovery of antibiotics as broad spectrum chemotherapeutics for bacteria greatly enhanced our ability to fight off and control bacterial diseases. However, commiserate with the general use of antibiotics, bacteria have responded by developing resistance to these important and ubiquitous compounds. One strategy employed to enhance resistance against microbial pathogens is to directly stimulate the host immune response and allow natural, host mediated, killing mechanisms to control microbial infections. These novel immunotherapeutics could be used independently or in context of antibiotic therapy to aid in clearance of bacteria. In turn this would allow for a decrease the amount of time antibiotics should be administered, an increase in the time before antibiotics must be administered, and/or lower dosages of antibiotics required for complete clearance of the bacterium.

Here we describe a novel, broad spectrum antimicrobial immunoprophylaxis consisting of cationic DNA liposome complexes (CLDC) and crude membrane preparations (MPF) derived from *F. tularensis* that effectively limited replication of virulent *F. tularensis, B. pseudomallei, Y. pestis* and *B. abortus* in human and mouse macrophages *in vitro*. Importantly, administration of CLDC+MPF prior to pulmonary infection with *F. tularensis* also contributed to survival in mice. The mechanism of protection mediated by CLDC+MPF was, in part, dependent on the induction of reactive oxygen and nitrogen species *in vivo* and *in vitro*.

Previous reports have shown that either CpG oligodeoxynucleotides (ODN) or CLDC alone can protect against lethal infections with attenuated strains of *F. tularensis*, e.g. LVS [27], [28], [29]. However, neither of these therapeutics have been able to protect animals from death following SchuS4 infection [27], [29]. We confirmed and extended these results by demonstrating that regardless of the route of time CLDC was administered prior to challenge, this reagent could not decrease the number of mortalities among SchuS4 infected mice. In fact, in our hands injection of CLDC 1 or 2 days prior to SchuS4 challenge exacerbated disease as indicated as a decrease in the mean time to death (Table 3). Our results do slightly differ from those reported by Troyer et al, but are closer in agreement with the report by Rozak et al in which administration of CpG ODN less than 24 hours prior to infection exacerbated disease [27], [29]. In the experiments reported by Troyer et al., addition of DNA to cationic liposomes was performed under standard laboratory conditions with no obvious means to monitor quality control. Further, there was no indication of endotoxin levels present in the preparations of DNA. Addition of other TLR agonists to cationic liposomes enhances their immunogenicity [30]. Thus, contaminating endotoxin could have increased and/or changed the inflammatory response in the Troyer study resulting in a different mean time to death. The CLDC used in the study presented herein were produced under strict GMP laboratory conditions and underwent a battery of quality control assays prior to use to insure consistency from lot to lot. Thus, it is possible that minor variations in CLDC preparations used in the Troyer study could account for the 1.4 day extension in mean time to death among SchuS4 infected mice.

It is not clear why CLDC exacerbated infection in vitro and in vivo. One possibility is that activation of macrophages with CLDC increases their phagocytic capability without eliciting effective killing mechanisms. Indeed, immediately after infection CLDC treated cells had significantly more intracellular bacteria compared to untreated cells (p<0.05) (Table 2). MPF alone also activates macrophages and increased uptake of SchuS4. Thus, it is possible that the exacerbation of infection observed in macrophages treated with either CLDC or MPF alone may be a direct result of increased phagocytosis in the absence of effective killing.

In contrast to exacerbation of infection in mice treated with CLDC alone, we observed a small increase in the mean time to death among animals treated with CLDC alone 3 days prior to infection (Table 3). Similarly, CpG has been noted to increase the mean time to death of SchuS4 infected mice by 1 day if delivered 2 days prior to infection [29]. This suggested CLDC and CpG alone could contribute toward controlling SchuS4 infection when delivered at the appropriate time before infection. CLDC does contain CpG ODN sequences. However, these sequences are not required for CLDC to exert protective effects against infections in vivo [7]. The induction of inflammatory responses by CLDC which do not contain CpG motifs may be attributed to cellular recognition of bacterial DNA. Although TLR9 represents an important receptor for recognition of CpG motifs present in bacterial DNA, it is not the only host receptor capable of detecting prokaryotic DNA. For example, DAI is a cytosolic receptor capable of detecting bacterial DNA that does not contain CpG motifs and can trigger immune responses in mammalian cells in a TLR9 independent manner [31]. Thus, the immunogenicity of CLDC cannot be completely attributed to the presence of CpG.

One of the major components of MPF is LPS. LPS from LVS lacks properties typically associated with endotoxin, e.g. stimulation of pro-inflammatory cytokines (25). However, injection of LVS LPS can protect animals from lethal LVS infections. Thus, we tested LPS from both LVS and SchuS4 for protective efficacy against pulmonary tularemia. Surprisingly, neither LPS preparation was able to protect animals from death following intranasal SchuS4 infection (Table 4). Administration of Francisella LPS in CLDC did modestly increase the mean time to death in SchuS4 infected animals, but these animals eventually succumbed to infection (Table 4). This suggested that Francisella LPS alone is not the bacterial antigen contributing to the protective effects of CLDC+MPF. Another bacterial ligand that may contribute to the protective efficacy of CLDC+MPF is peptidoglycan or one of its precursors, i.e. muramyl dipeptide (MDP) or tracheal cytotoxin (TCT). Both MDP and TCT can contribute toward the induction of nitric oxide [32], [33], [34], [35], [36]. Interestingly, MDP typically requires cells to be primed with IFN-γ or other immunostimulants in order to induce nitric oxide [35], [36]. We have not quantitated MDP or TCT present in our MPF preparations. However, it is tempting to speculate that either or both of these compounds may contribute to the protective efficacy of CLDC+MPF.

In both the *in vitro* and *in vivo* models pre-stimulation of macrophages before replication of SchuS4 began was required for killing of bacteria. *In vitro*, cells stimulated with CLDC+MPF 12 h after infection failed to significantly control Francisella replication (Figure S3). Similarly, administration of CLDC+MPF less than three days prior to pulmonary challenge failed to protect animals from lethal disease (CM Bosio, unpublished data). There are several explanations for this pre-stimulation requirement. First, the kinetics of SchuS4 intracellular replication on the level of individual cells following in vivo infection has not been defined. It is possible that following infection of macrophages in vivo SchuS4 does not undergo a lag phase prior to replication such as that observed among in vitro infected macrophages. Second, SchuS4 targets multiple cell types in vivo. In addition to macrophages, this bacterium also infects dendritic cells and epithelial cells at the outset of infection [37], [38]. It is not known if CLDC+MPF activates dendritic cells and epithelial cells in the same manner we have observed in macrophages. Longer stimulation of these cells may be required for adequate priming of killing mechanisms in these and/or neighboring cells.

The third possibility may be a requirement for activation of other host effector cells or molecules. It has been suggested that NK cells contribute to eradication of *F. tularensis* following pulmonary infections [39]. Interestingly, intravenous injection of CLDC results in accumulation of NK cells in the lungs which peaks three days after injection [40]. Data from our laboratory suggests that SchuS4 specific NK cells present in lungs of vaccinated mice are capable of controlling SchuS4 replication in this tissue (CM Bosio and RV Anderson, unpublished data). Thus, injection of CLDC+MPF three days prior to infection may allow the accumulation of SchuS4 specific NK cells capable of restricting bacterial replication in the lungs.

The requirement for pre-stimulation may also lie in the amount of time necessary for ROS and RNS to be activated. *F. tularensis* encodes genes that specifically interfere with production of reactive oxygen and nitrogen species [41]. In the absence of pre-activation, RNS and ROS generation in host macrophages is efficiently impeded by virulent *F. tularensis* [41]. Similarly, interference with induction of RNS and ROS as a mechanism to evade killing has also been reported for *Y. pestis* and *B. pseudomallei* [42], [43]. Thus, generation of adequate RNS and ROS by hosts cells prior to infection or replication of bacteria if host cells would be required for optimal control of infections with these virulent bacteria.

An additional explanation for the necessity of pre-stimulation of host cells lies in the mechanism by which RNS and ROS are generated. Reactive oxygen and nitrogen species are most effectively produced in response to several pro-inflammatory cytokines. For example, although neither TNF-α nor IFN-β can act alone to induce release of nitric oxide or hydrogen peroxide, these cytokines act synergistically with bacterial antigens to augment production of these two antimicrobial compounds [44]. Our data demonstrate that CLDC, and in some cases CLDC+MPF, induced significantly more TNF-α and IFN-β compared to untreated cells or cells exposed to MPF alone (Figure 4). Thus, it is possible that optimal stimulation of RNS and ROS was dependent on the generation of these, and perhaps other, proinflammatory cytokines which would require additional time for generation of ROS and RNS prior to infection.

Another important observation made in the studies presented herein was that the putative roles for ROS and RNS for control of *F. tularensis* were dramatically different in mouse and human macrophages. As described above, CLDC+MPF mediated killing in mouse macrophages was dependent on the generation of ROS and RNS (Figure 5A–C). This is in agreement with previous reports describing the contribution of these species for control of more attenuated strains of *F. tularensis* [15], [41], [45]. However, in human macrophages inhibition of RNS had no effect on the antibacterial activity mediated by CLDC+MPF on *F. tularensis* (Figure 5D and E). Rather, generation of ROS was essential for CLDC+MPF mediated killing of *F. tularensis*. In the past it was commonly believed that human cells could not produce RNS. Thus, one might assume that the dependency on ROS for CLDC+MPF mediated killing in human cells was due to an inherent inability of these macrophages to generate RNS. Indeed, soon after the description of cytokine induced nitric oxide in mouse cells, investigators attempted to reproduce the phenomenon in human macrophages. However, early studies in human cells did not recapitulate the potent nitric oxide response observed in mouse macrophages [46]. This led to the hypothesis that human cells did not express the product responsible for cytokine induced nitric oxide, iNOS. Since those early studies there have been a number of reports demonstrating the presence and function of iNOS in human cells [47]. We now understand that the inability of human macrophages to produce nitric oxide in response to cytokines alone was due to the nature of the cell type (as reviewed, [48]). For example, macrophages differentiated in vitro from resting peripheral blood monocytes of normal donors generally do not express iNOS. In contrast, macrophages differentiated from monocytes obtained from donors with chronic inflammatory disorders or currently battling infection readily express iNOS [49], [50]. Thus, under the appropriate conditions human macrophages, like mouse macrophages can induce RNS. As shown in the present manuscript, IFN-γ induced RNS is capable of controlling SchuS4 replication in human macrophages, thus confirming the ability of human cells to generate effective RNS responses. Therefore, the role for ROS in CLDC+MPF mediated killing of SchuS4 in human macrophages is not due to the inability of the cells to generate RNS, but is a unique feature of CLDC+MPF stimulation of this cell type.

Understanding and identifying the different requirements and redundancy for RNS and ROS in mouse and human cells is important for several reasons. First, identification of the mechanisms by which human and mouse cells control infections with virulent bacteria is essential for monitoring the potential effectiveness of novel drugs. Second, identification of the specific killing pathways will also aid in development of other novel therapeutics. Lastly, understanding the different requirements for bacterial killing in human and mouse macrophages may reveal new pathways used by host cells to effectively combat invading pathogens. As described above, unlike stimulation with IFN-γ, CLDC+MPF revealed a difference in the mechanism by which human and mouse macrophages control virulent *F. tularensis*. Thus, this reagent may also serve as a useful tool to dissect the relative roles of these pathways in the effective control of infections with virulent bacteria. This in turn may result in superior immunoprophylaxis and therapeutics for infections mediated by diverse groups of bacteria.

## Materials and Methods

### Ethics statement

Human blood cells were collected from anonymous volunteers under a protocol reviewed and approved by the NIH Clinical Center Institutional Review Board. Signed, informed consent was obtained from each donor acknowledging that their donation would be used for research purposes by intramural investigators throughout NIH.

### Bacteria

*Francisella tularensis* strain SchuS4 was kindly provided by Jeannine Peterson, Ph.D. (Centers for Disease Control, Fort Collins, Colorado), *F. tularensis* strain LVS was provided by Jean Celli, Ph.D. (Rocky Mountain Laboratories, Hamilton, Montana). SchuS4 and LVS were cultured in modified Mueller-Hinton (MMH) broth at 37°C with constant shaking overnight, aliquoted into 1 ml samples, frozen at −80°C and thawed just prior to use as previously described [37]. Frozen stocks were titered by enumerating viable bacteria from serial dilutions plated on modified Mueller-Hinton agar as previously described [51], [52]. The number of viable bacteria in frozen stock vials varied less than 5% over a 10 month period.

*Yersinia pestis* strain 195/P expressing GFP was provided by B. Joseph Hinnebusch, Ph.D. (Rocky Mountain Laboratories, Hamilton, Montana). 195/P-GFP was cultured overnight at 21°C in BHI broth followed by subculture at 37°C as previously described [43]. Bacterial titer was estimated by optical density of the culture at 600nm. Inoculum titers were confirmed following enumeration of viable bacteria from serial dilutions plated on blood agar plates as previously described [43].

*Burkholderia pseudomallei* strain DD503 expressing GFP was provided by David DeShazer, Ph.D. and Mary Burtnick, Ph.D. (USAMRIID, Fort Detrick, MD and University of South Alabama, Mobile AL, respectively). DD503-GFP was cultured in LB broth overnight at 37°C. Three hours before use, DD503-GFP was diluted 1∶25 in TSBDC culture medium [53] and incubated at 37°C. Bacterial titer was estimated by optical density of the culture at 600nm. Immediately prior to use bacteria were diluted in tissue culture medium and added to cells as described below. Inoculum titers were confirmed following enumeration of viable bacteria from serial dilutions plated on LB agar as previously described [54].

*Brucella abortus* strain 2308 expressing GFP was kindly supplied by Jean Celli, Ph.D. (Rocky Mountain Laboratories, Hamilton, MT). GFP-*B. abortus* was cultured on Tryptic Soy agar (TSA) plates for 48 h at 37°C. Individual colonies were then transferred to Tryptic Soy Broth (TSB) and bacteria were cultured overnight at 37°C with constant shaking. The number of bacteria present on broth cultures was determined by OD 600nm. Actual numbers of viable bacteria were confirmed by plating an inoculum on TSA plates as previously described [55].

### Generation of MPF, LPS and CLDC+MPF

LVS was grown in MMH broth as described above. Following overnight culture, LVS was centrifuged for 15 minutes at 8000×g. The resulting pellet was resuspended in breaking buffer (50 mM Tris/HCl, 0.6 µg/ml DNase, 0.6 µg/ml RNase, 1 mM EDTA [all from Sigma] and 1 Complete EDTA free tablet [Roche]) and the bacteria were centrifuged again for 15 minutes at 8000×g. Pelleted bacteria were then resuspended in breaking buffer. To break open LVS, the bacteria were added to Fast Prep Lysing Matrix B tubes and processed in a FastPrep24 (MPBio) for 10 cycles of 45 seconds with 2 minute rest periods on ice in between each cycle. The resulting slurry was then centrifuged at 10,000 rpm for 10 minutes. The supernatant was collected and centrifuged twice at 100,000×g for 4 h. The pellet was resuspended in buffer containing 50 mM Tris/HCl, 1 mM EDTA and dialyzed against PBS using 3000 MW cutoff Slide-A-Lyzer cassettes (Pierce). Protein concentration of LVS membrane protein fraction (MPF) was determined using a BCA Protein Assay Reagent Kit according to the manufacturer's instructions. Endotoxin levels were determined using Limulus Amebocyte Lysate (LAL) assay. Endotoxin levels were <0.1 EU/µg of protein. MPF was then aliquoted, irradiated to render it sterile, and stored at -80°C. SchuS4 LPS was generated as previously described [23]. LVS LPS was obtained from the BEI Resources (Manassas, VA).

CLDC was provided by Juvaris Biotherapeutics. Formulation of CLDC has been previously described [56]. CLDC was prepared by Juvaris Therapeutics under good manufacturing conditions (GMP). Briefly, DOTIM∶cholesterol liposomes were combined to form a liposome intermediate. pMB75.6 plasmids were generated from the non-pathogenic strain of *E. coli* DH5α. The plasmid contains the following elements: (i) the cytomegalovirus immediate early (CMV-IE) promoter/enhancer, (ii) a polyadenylation signal derived from simian virus 40 (SV_40_), (iii) a kanamycin-resistance gene, and (iv) an origin of replication (pUCori/f1ori). There are no genes expressed by the plasmid. pMB75.6 plasmids were added to liposomes at a ratio of 9.8∶1 (lipid∶DNA). CLDC were aliquoted, lyophilized, and stored at 4°C. Endotoxin levels were determined using Limulus Amebocyte Lysate (LAL) assay. Endotoxin levels of CLDC were <0.1 EU/mg DNA.

Immediately prior to use, CLDC was hydrated using 500 µl endotoxin free, water (Cape Cod Associates Incorporated, E. Falmouth, MA). CLDC were allowed to rehydrate for approximately 5 minutes at room temperature. CLDC were then diluted 1∶3 in 5% dextrose water (D5W; Baxter Healthcare, Deerfield, IL). For *in vitro* experiments, MPF was thawed, vortexed and added to CLDC at a final concentration of 3.52 µg/ml. As indicated, MPF and CLDC were also tested individually. In these experiments MPF was diluted in 5% dextrose water to 3.52 µg/ml. All CLDC, MPF and CLDC+MPF mixtures were added to cells in a volume of 50 µl/well and were used immediately following preparation.

For *in vivo* experiments, MPF or LPS was thawed, vortexed, and diluted in 5% dextrose water. In experiments testing MPF or LPS alone, MPF and LPS were diluted to 50 µg/ml. In experiments testing combination of CLDC and MPF or LPS, the reagents were added to CLDC at a final concentration of 50 µg/ml. In these experiments MPF was diluted in 5% dextrose water to 50 µg/ml. All CLDC, MPF and CLDC+MPF mixtures were used immediately following preparation. Mice were injected with 200 µl of prepared CLDC+MPF.

### Culture of bone marrow derived macrophages

Bone marrow derived macrophages were generated as previously described [51] with the exception that cells were differentiated into macrophages in tissue culture plates with or without glass coverslips in the presence of 10 ng/ml recombinant murine M-CSF (Peprotech, Rocky Hill, NJ). All cells were used on day 6 of culture.

### Generation of human monocyte derived macrophages

Human monocyte derived macrophages were differentiated from apheresed peripheral blood monocytes as previously described [57]. Briefly, apheresed monocytes were enriched using Ficoll-paque (GE Healthcare). CD14^+^ progenitor cells were enriched via negative selection using Dynabeads MyPure Monocytes Kit for untouched human cells per manufacturer's instructions (Invitrogen). Cells were resuspended at 3×10^5^/ml in cRPMI supplemented with 10 ng/ml M-CSF (Peprotech) plated at 1 ml/well in 24-well plates with or without glass coverslips and incubated at 37°C/5%CO_2_. On day 2 and 5 of culture medium was replaced with cRPMI supplemented with 10 ng/ml M-CSF. All cells were used on day 6 of culture. Human blood cells were collected from anonymous volunteers under a protocol reviewed and approved by the NIH Clinical Center Institutional Review Board. Signed, informed consent was obtained from each donor acknowledging that their donation would be used for research purposes by intramural investigators throughout NIH.

### Treatment and infection of macrophages

Macrophages were treated with CLDC, MPF or CLDC+MPF, prepared as described above, 18 h prior to infection with bacteria. As indicated, 1 or 6 hr prior to addition of CLDC+MPF human and mouse macrophages were pretreated with 10 mM (human) or 3 mM (mouse) N-acetyl-L-cysteine (NAC) (Sigma), 3 mM (human) or 1 mM (mouse) L5-[imino(methylamino)methyl]-L-ornithinemonoacetate (L-NMMA), (Caymen Chemicals), respectively.

Following treatments described above, macrophages were infected with either *F. tularensis* SchuS4 (MOI = 50), *B. abortus* (MOI = 50), *B. pseudomallei* (MOI = 1), or *Y. pestis* (MOI = 2). Briefly, medium was removed and reserved, and then *F. tularensis* was co-incubated with macrophages at 37°C in 7% CO_2_ for 1.5 h followed by treatment with gentamicin (Invitrogen) at 500 µg/ml for 1 h. Then, cultures were washed extensively and reserved medium was replaced. The infection inoculum was confirmed by plating serial dilutions of stock *F. tularensis* on MMH agar plates immediately prior to addition to cell cultures.

*B. pseudomallei* was co-incubated with macrophages at 37°C in 5% CO_2_ for 1 h followed by addition of kanamycin (Invitrogen) at 250 µg/ml for the remainder of the experiment.

*Y. pestis* was co-incubated with macrophages at 21°C in 5% CO_2_ for 1 h followed by treated with gentamicin at 80 µg/ml for 1 h. Then, cultures were washed extensively and reserved medium supplemented with 8 µg/ml gentamicin was replaced.

Macrophages were infected with *B. abortus* as previously described [55]. At the indicated time points, cells were either lysed for enumeration of intracellular bacteria or isolation of RNA, or were fixed with 3% paraformaldehyde in PBS for 20 min at 37°C/5%CO_2_ prior to analysis for bacteria as described below.

### Enumeration of bacteria

At the indicated time points, medium was removed and cells were washed extensively. Then, cells were lysed following incubation in sterile water. Cell lysates were immediately serially diluted and plated on either MMH, LB, blood, or TSA agar plates. Agar plates were incubated at 37°C/7% CO_2_ for 48–72 h for enumeration of bacterial colonies.

### Fluorescence microscopy

Macrophages were grown on coverslips, treated, and infected with SchuS4, GFP-*B. pseudomallei*, GFP-*Y. pestis* or GFP-*B. abortus* as described above. Cells were fixed in 3% paraformaldehyde for 20 minutes at 37°C/5%CO_2_. Cells were washed with PBS and stained for LAMP-1 as previously described [10], [57]. SchuS4 was detected using Alexa Fluor488 goat conjugated anti-*F. tularensis* (US Biological, Swampscot, MA) as previously described [57]. Samples were observed on a Carl Zeiss (Thornwood, NY) Axio Imager.M1 epifluoresence microscope for quantitative analysis. Approximately 75–100 cells/field and a minimum of three fields per coverslip were analyzed for presence of intracellular bacteria. Percent of infected cells was calculated as follows: (number infected cells/total number of cells) ×100.

Confocal images of 1,024×1,024 pixels were acquired and assembled using Adobe Photoshop CS2 software (Adobe Systems, San Jose, CA).

### Quantitative PCR analysis

RNA was isolated and converted to cDNA using RT^2^ qPCR Grade RNA Isolation Kit and RT^2^ First Strand Kit (both from SA Biosciences, Frederick, MD) according to manufacturer's instructions. Samples were assessed for ROS and RNS associated genes expression using nitric oxide RT^2^ Profiler PCR Arrays for mouse and human per manufacturer's instructions (SA Biosciences). Gene expression was quantitated using RT^2^ Profiler PCR Array Data Analysis Software (SA Biosciences). Accession numbers for genes in which expression was increased or decreased compared to untreated controls are listed in Table S1.

### Mice

Specific-pathogen-free, 6–8 week old male C57Bl/6 mice (wild type) (n = 10/group) were purchased from Jackson Laboratories (Bar Harbor, MI). nos2/phox gp91 deficient mice (nos2/gp91^−/−^) mice were bred at the NIAID/Rocky Mountain Laboratories. All research involving animals was conducted in accordance with Animal Care and Use guidelines and animal protocols were approved by the Animal Care and Use Committee at RML.

### Treatment and infection of mice

CLDC, MPF and CLDC+MPF were prepared as described above. At the time points indicated, mice were injected intravenously (i.v.), intraperitoneally (i.p.) or subcutaneously (s.c.) with 200 µl of each preparation prior to challenge. For intranasal (i.n.) administration mice were anesthetized via intraperitoneal injection with 100 µl of ketamine (12.5 mg/ml) + xylazine (3.8 mg/ml) solution and the reagents were delivered in a total volume of 25 µl evenly distributed between the nares at the indicated time points prior to challenge. Then, mice were anesthetized via intraperitoneal injection with 100 µl of ketamine (12.5 mg/ml) + xylazine (3.8 mg/ml) solution and intranasally infected with 25 CFU SchuS4 diluted in a final volume of 25 µl of PBS. Inoculating doses were confirmed by plating inoculum on MMH agar. This inoculum routinely results in 100% mortality and a mean time to death of 5 days following infection in naive animals.

### Statistical analysis

For *in vitro* studies, statistical differences between two groups were determined using an unpaired student t test with the significance set at p<0.05. For comparison between three or more groups, analysis was done by one-way ANOVA followed by Tukey's multiple comparisons test with significance determined at p<0.05. For *in vivo* studies, significance in survival was assessed using log-rank (Mantel Cox) test with significance set at p<0.05.

## Supporting Information

Figure S1 CLDC+MPF does not induce cell death. Macrophages were treated with D5W (untreated), CLDC, MPF or CLDC+MPF as described in Figure1. Eighteen hours after treatment cell death was assessed by uptake of trypan blue. CLDC+MPF did not induce cell death that was significantly different from untreated, CLDC or MPF treated controls. Data is representative of three experiments of similar design. Error bars represent SEM.
(1.00 MB TIF)

Figure S2 CLDC+MPF elicits degradation of SchuS4 in macrophages. Human macrophages were treated with D5W or CLDC+MPF and were either left uninfected (A and D) or infected with SchuS4 (B–C and E–F) as described in Figure 1. Twenty-four hours after infection cells were prepared for analysis by transmission electron microscopy. Following infection, D5W treated cells contained intact SchuS4 (B and C) whereas vacuoles present in CLDC+MPF treated cells consisted predominantly of degraded bacteria (E and F). White arrows indicated bacteria. C and F are the magnified areas indicated by the black box in B and E, respectively. A, B, D, and E are depicted at 4000× magnification. C and F are depicted at 12000× magnification. Data is representative of two experiments of similar design.
(2.04 MB TIF)

Figure S3 CLDC+MPF controls SchuS4 replication when added within 12 hours of infection. Human macrophages were treated with D5W (untreated) or CLDC+MPF 18 h prior to infection (−18), 4 h after infection (+4) or 12 h after infection (+12). Twenty four hours after infection intracellular replication of F. tularensis was monitored by microscopy. * = p<0.001 compared to D5W and +12 h treated cells. Data is representative of two experiments of similar design. Error bars represent SEM.
(0.07 MB TIF)

Figure S4 Induction of RNS and ROS genes by IFN-gamma and CLDC+MPF. Mouse and human macrophages were treated with D5W, IFN-gamma or CLDC+MPF. After 12 h gene expression was monitored by quantitative RT-PCR. Change in the expression of the indicated genes is represented as fold change over D5W treated controls.
(0.05 MB TIF)

Table S1 Gene accession numbers.
(0.01 MB DOCX)
