# Supplementary figures and images for: Effective, Broad Spectrum Control of Virulent Bacterial Infections Using Cationic DNA Liposome Complexes Combined with Bacterial Antigens
Source: PLoS Pathog. 2010 May 27;6(5):e1000921. doi: 10.1371/journal.ppat.1000921 (PMC2877747; doi:10.1371/journal.ppat.1000921)

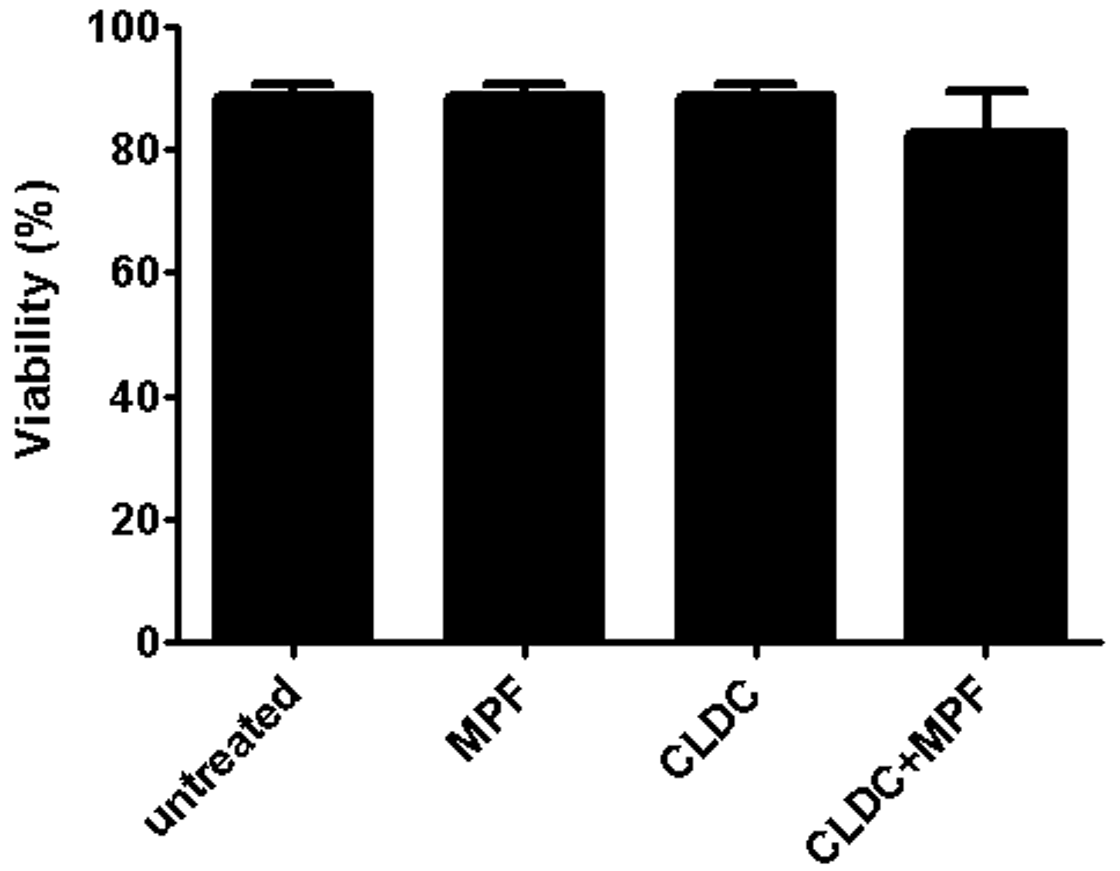

Supplement: Figure S1 — CLDC+MPF does not induce cell death. Macrophages were treated with D5W (untreated), CLDC, MPF or CLDC+MPF as described in Figure1. Eighteen hours after treatment cell death was assessed by uptake of trypan blue. CLDC+MPF did not induce cell death that was significantly different from untreated, CLDC or MPF treated controls. Data is representative of three experiments of similar design. Error bars represent SEM. (1.00 MB TIF) [file ppat.1000921.s001.tif]

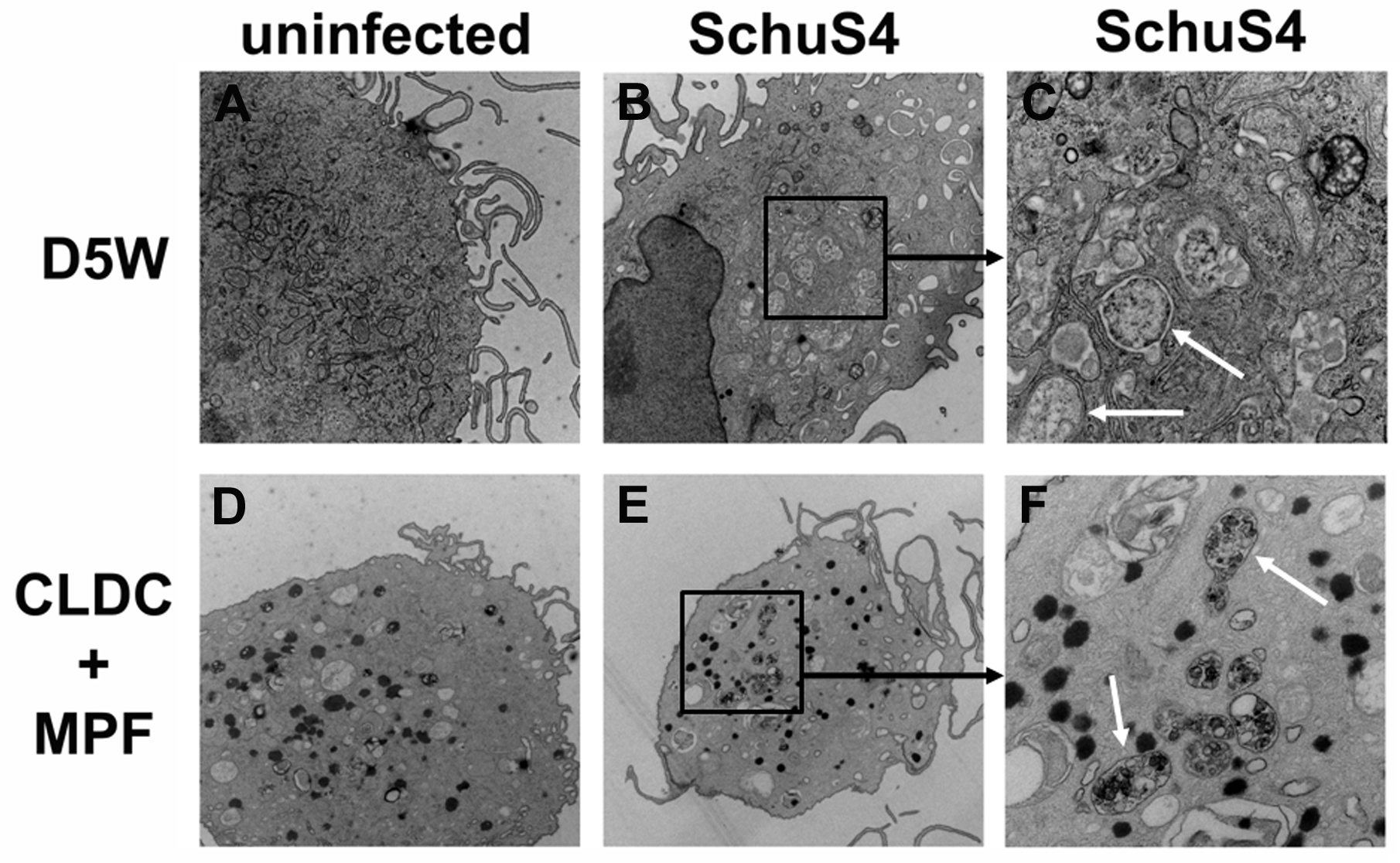

Supplement: Figure S2 — CLDC+MPF elicits degradation of SchuS4 in macrophages. Human macrophages were treated with D5W or CLDC+MPF and were either left uninfected (A and D) or infected with SchuS4 (B–C and E–F) as described in Figure 1. Twenty-four hours after infection cells were prepared for analysis by transmission electron microscopy. Following infection, D5W treated cells contained intact SchuS4 (B and C) whereas vacuoles present in CLDC+MPF treated cells consisted predominantly of degraded bacteria (E and F). White arrows indicated bacteria. C and F are the magnified areas indicated by the black box in B and E, respectively. A, B, D, and E are depicted at 4000× magnification. C and F are depicted at 12000× magnification. Data is representative of two experiments of similar design. (2.04 MB TIF) [file ppat.1000921.s002.tif]

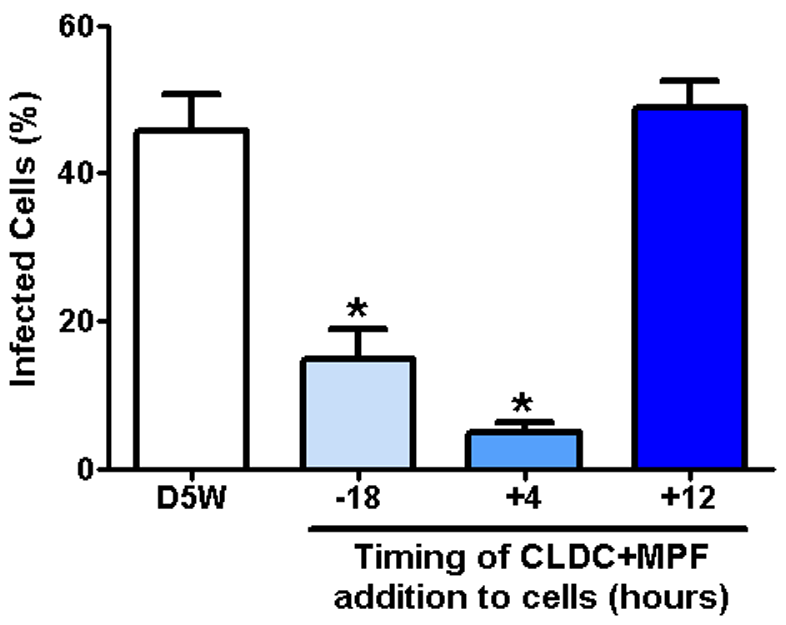

Supplement: Figure S3 — CLDC+MPF controls SchuS4 replication when added within 12 hours of infection. Human macrophages were treated with D5W (untreated) or CLDC+MPF 18 h prior to infection (−18), 4 h after infection (+4) or 12 h after infection (+12). Twenty four hours after infection intracellular replication of F. tularensis was monitored by microscopy. * = p<0.001 compared to D5W and +12 h treated cells. Data is representative of two experiments of similar design. Error bars represent SEM. (0.07 MB TIF) [file ppat.1000921.s003.tif]

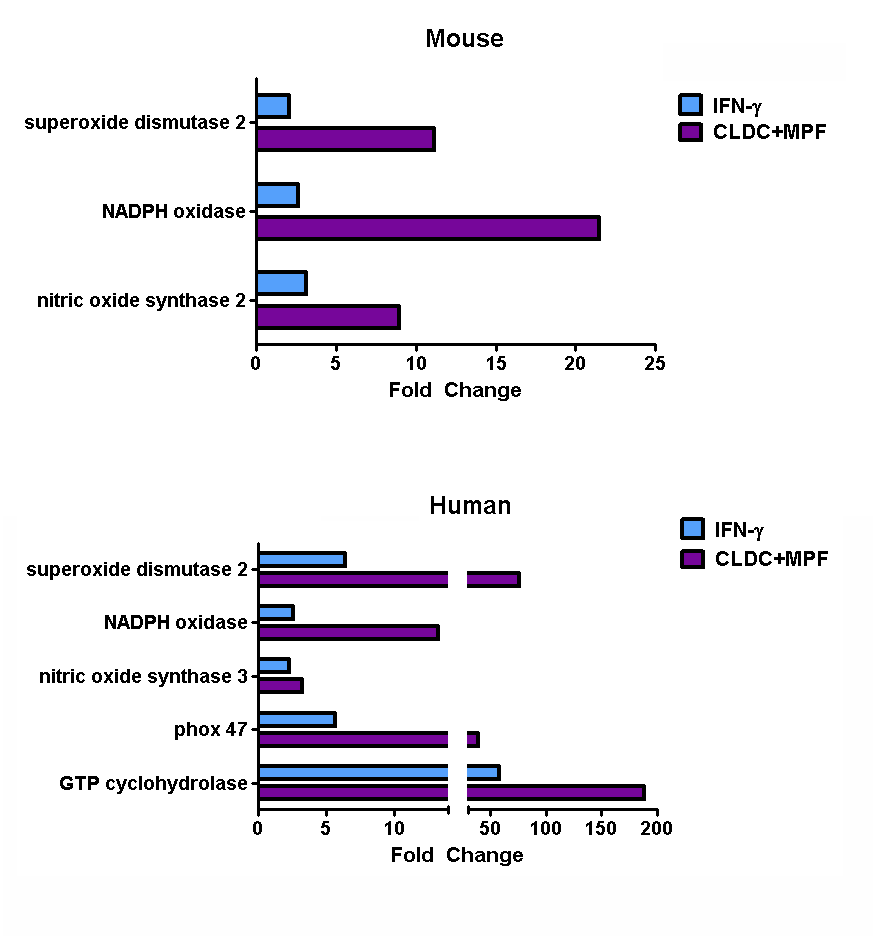

Supplement: Figure S4 — Induction of RNS and ROS genes by IFN-gamma and CLDC+MPF. Mouse and human macrophages were treated with D5W, IFN-gamma or CLDC+MPF. After 12 h gene expression was monitored by quantitative RT-PCR. Change in the expression of the indicated genes is represented as fold change over D5W treated controls. (0.05 MB TIF) [file ppat.1000921.s004.tif]
