# Supplementary material for: Effective, Broad Spectrum Control of Virulent Bacterial Infections Using Cationic DNA Liposome Complexes Combined with Bacterial Antigens
Source: PLoS Pathog. 2010 May 27;6(5):e1000921. doi: 10.1371/journal.ppat.1000921 (PMC2877747; doi:10.1371/journal.ppat.1000921)
Supplement: Table S1 — Gene accession numbers. (0.01 MB DOCX) [file ppat.1000921.s005.docx]

Supplemental Table I. Accession numbers for genes reported in Figure 3

Mouse

| **Gene** | **Symbol** | **GenBank** |
| --- | --- | --- |
| superoxide dismutase 3 | Sod3 | NM_011435 |
| superoxide dismutase 2 | Sod2 | NM_013671 |
| NADPH oxidase 1 | Nox1 | NM_172203 |
| nitric oxide synthase 2 | Nos2 | NM_010925 |

Human

| **Gene** | **Symbol** | **GenBank** |
| --- | --- | --- |
| superoxide dismutase 2 | SOD2 | NM_000636 |
| NADPH oxidase | NOX5 | NM_024505 |
| nitric oxide synthase 2A | NOS2A | NM_000625 |
| neutrophil cytosolic factor 1 (phox 47) | NCF1 | NM_000265 |
| GTP cyclohydrolase | GCH1 | NM_000161 |
